# Faster but less accurate: An explorative study on the effects of three weeks of ketogenic diet on cognitive functions in undergraduate students

**DOI:** 10.1371/journal.pone.0338877

**Published:** 2026-01-14

**Authors:** Gianluigi Serio, Consiglia Pacelli, Claudia Piccoli, Nazzareno Capitanio, Giuseppe Cibelli, Anna Antonia Valenzano, Francesca Landini, Leonardo Carlucci, Paola Palladino

**Affiliations:** 1 Department of Humanities, Literature and Cultural Heritage, University of Foggia, Italy; 2 Department of Clinical and Experimental Medicine, University of Foggia, Italy; University of Rijeka Faculty of Health Studies: Sveuciliste u Rijeci Fakultet zdravstvenih studija, CROATIA

## Abstract

The ketogenic diet (KD) is a low-carbohydrate diet that induces and sustains a ketosis state and minimizes somatic glucose levels. Several psychological studies have described the positive effects of ketosis on cognitive functions for a wide range of neuropsychiatric conditions (e.g., Alzheimer’s disease; epilepsy), leading to greater interest in the KD today. However, the psychological and cognitive effects of inducing ketosis via diet remain unclear, especially in healthy people. From an initial pool of thirty participants, eight undergraduate students performed a cognitive assessment before (baseline) and after three weeks (follow-up) of an isocaloric ketogenic diet. Several neuropsychological measures and psychometric tests have been administered to investigate psychological chronotype, sleep quality, eating habits, anxiety and cognitive components of attention, inhibition, and memory. Non-parametric Bayesian analysis showed that the ketogenic diet affected cognitive functions. Participants performed cognitive tests faster at follow-up than at baseline, showing improvements in visual-motor cognitive and processing speed components. However, they were less accurate on working memory tasks, suggesting a decreasing performance of higher cognitive functions. Finally, no differences in anxiety levels were found between baseline and follow-up. The results could have significant implications for identifying specific cognitive models of students based on specific lifestyle habits and nutritional patterns, allowing the implementation of targeted interventions to improve university learning conditions.

## Introduction

### Background

The ketogenic diet is a very high-fat and low-carbohydrate diet that induces and sustains a ketosis state and minimizes somatic glucose levels in the body without causing caloric restriction or malnutrition [1–4]. The hallmark of the KD is the production of ketone bodies (mainly β-hydroxybutyrate, acetoacetate, and acetone) from the oxidation of fatty acids in the liver, which provides an alternative substrate to glucose for energy utilization [5,6]. The ketogenic diet (KD) is primarily characterized by a very low carbohydrate intake, typically less than 50 g per day, and often as low as 20–30 g/day in therapeutic settings, which shifts metabolism toward ketone body production [7,8]. Although fat intake is usually increased, the defining feature of KD is carbohydrate restriction rather than high fat per se [9]. Ketone bodies are nutritional biomarkers carrying antioxidative processes that promote mitochondrial integrity and support neuro synaptic function [10,11].

Positive effects of the KD have been described in the treatment of epilepsy in both adults and children [12–15]. It has also been suggested that the KD and the consequent increased circulating levels of ketone bodies could prevent age-related cognitive decline [16] by improving – among others – central nervous system insulin resistance [17,18]. Indeed, several recent studies have reported that ketosis could be effective in reducing cognitive decline in patients with Alzheimer’s disease and people with Mild Cognitive Impairment (MCI) [10,19,20].

The positive effects of ketosis in clinical populations have gained increased interest in the ketogenic diet [21,22], raising questions about its possible benefits in healthy populations. Despite this, studies with typical participants are still few and challenging to interpret due to methodological limits and differences. Some studies have explored the effect of medium-chain triglycerides (MCTs – a dietary supplement that promotes the production of ketone bodies) on cognitive functions in healthy older and young adults [23–25]. In detail, Ota and collaborators [23] showed that improvements in cognitive tasks (digit span, visual memory span, Letter-Number sequencing Test, Trail-Making Test) following a single ketogenic meal containing 20 mg of MCTs, occurred mainly in older adults who had lower cognitive scores at baseline. Despite the small experimental sample (N = 19), it should be underlined that the improvement in cognitive functions was correlated to the level of ketones in the plasma with higher ketone levels associated with greater cognitive improvement [23]. In Ashton et al. [24], a group of undergraduate students (N = 30) were split into three groups: placebo, 12 g MCT/day, and 18 g MCT/day. Participants were tested for five consecutive weeks on the following tasks: Trail-Making Test, Digit Span, Spatial Span, Cover Shift of Attention, and Rapid Visual Information Processing. The participants who took MCT (both 12 mg and 18 mg), compared to the placebo group, improved in the Trail-Making Test, in the digit span (forward/backward), and in the Spatial Span (backward). It is important to note that performance was slightly dependent on dosage. In particular, the 18 g MCT/day group’s performance in the Trial-Making Test (A) from the third week was significantly better than that of the placebo group. Similarly, the 12 g MCT/day group performed better from the fourth week than the control group [24]. In Yomogida et al. [25], a group of healthy older adults (N = 20) performed N-back and Go-Nogo tasks during functional magnetic resonance imaging in a double-blind-placebo-controlled study. Results showed that a single MCT meal improved performance on the N-back task than a placebo meal. However, when participants were divided into two sub-groups based on the global level of cognitive function (higher or lower) assessed by the Mini-Mental State Examination (MMSE), the effects of MCT differed depending on the cognitive task. Indeed, the higher performance group showed improvement in the N-back task (indicating better working memory). In comparison, the lower performance group showed improvement in the Go-Nogo task (indicating better inhibitory control) after the MCT meal rather than after the placebo meal. The dorsolateral prefrontal cortex (dlPFC) showed smaller task-induced fMRI BOLD signal increases after MCT meals rather than a placebo. This suggests that the ketone bodies were consumed as extra brain energy, which underlies the better performance. Finally, when the authors considered the entire sample, performance on working memory was significantly better after MCT above all in the participants with smaller grey matter volume in the left dlPFC. Authors hypothesized that the participants with relatively reduced neurons benefited more from the extra energy source supplied by ketone bodies [25].

The studies mentioned above would suggest that MCTs alone might improve cognitive functions, by producing ketone bodies without the need for a traditional ketogenic diet. These effects are likely due, at least in part, to improved ketosis-induced mitochondrial metabolism [26].

### Ketogenic diet and cognitive functions

Despite the positive effects of diet on psychological and cognitive functions [27,28], it can also have adverse effects on cognitive functions [29–31], on mood (i.e., depression) and self-esteem [32], as well as having been associated with increased stress responses [33]. A plausible explanation for the negative cognitive effects of dieting concerns participants’ anxiety and worries about dieting, which can impair cognition by interfering, for example, with working memory capacity [34]. The food-related ruminative worries characteristic of dietary restraint and dieting [35], combined with the increased sensations of hunger characterized by dieters, are hypothesized to be sufficient to increase cortisol secretions and negatively influencing the working memory processes [36]. Furthermore, it has been highlighted that dietary supervision and adherence to specific dietary patterns are critical factors in determining cognitive outcomes [37,38].

It is important to note that studies investigating cognitive functions concerning diet displayed methodological differences regarding the population target investigated (e.g., old adults, young adults, or overweight people) and the type of diet involved (e.g., hypo-/iso-caloric, nutrient composition). Studies have only partially investigated the different components of cognitive functions, with tasks that were too simple for healthy participants. Moreover, studies have generally poorly controlled for psychological factors such as anxiety, chronotype, and sleep quality. Different macronutrients and specific diet programs have been associated with distinct cognitive outcomes (i.e., both improvements and worsening in cognitive functions), and meal composition can be more influential than meal size or timing, mainly when substantial nutritional variations from habitual meals are encountered [34,37,39,40]. These shortcomings make it difficult to compare results and make them less generalizable. In this regard, only a few studies have investigated cognitive functions in healthy participants during ketogenic diet regimes.

In a study by D’anci and Coll. [41], the authors tested the cognitive functions (visuospatial memory, memory span, and vigilance) of a group of women (N = 19) who chose to undergo one of two weight-loss diets: a low-carbohydrate diet (N = 9) or a diet with macronutrient proportions typically recommended by the Academy of Nutrition and Dietetics (AND; N = 10). Results showed that the low-carbohydrate diet group had faster reaction times during the attentional vigilance task than the AND diet group, suggesting better-sustained attention in the low-carbohydrate diet group. However, the low-carbohydrate diet group performed worse on memory-based tasks (Reverse Digit Span Task, short and long-term map-recall) than the AND diet group. On the contrary, performance on the less cognitively demanding Forward Digit Span was not affected. Notably, the weight loss over the three weeks of diet was less than 2 kg and was similar between the two diet groups [41]. It is important to note that this study [41] did not employ isocaloric control groups, and therefore, the observed results may reflect differences in energy intake or weight loss in addition to ketosis. More recent randomized, isocaloric crossover trials [42,43] have addressed this issue by directly isolating the effects of macronutrient composition from those of total energy intake. In another study, Iacovides et al. [42] tested cognitive functions (vigilance, visual learning, memory, working memory, and executive functions) of a small group of young adults (N = 11) undergoing the ketogenic diet and a high-carbohydrate diet. The authors reported that only processing speed was marginally faster following the KD than following the high-carbohydrate diet in the Two Back Test and the Groton Maze Learning Test, while participants were marginally slower in the Identification task after the KD than the high-carbohydrate diet [42]. Similarly, Shaw et al. [43] reported no effect of the KD on cognitive function (vigilance, sustained attention, and working memory), mood, and sleep in a small group of military personnel (N = 8) compared to a carbohydrate-based diet [43]. However, in another study with a small group of military personnel (N = 7), a KD demonstrated beneficial effects on cognitive performance (vigilance, sustained attention, and working memory) during thirty-six hours of extended wakefulness compared with a carbohydrate-based diet [44].

Indeed, the expression of individual cognitive potential can be affected significantly by the interaction between physical and psychological fatigue [44,45], quality of sleep [46], anxiety [47], and psychological chronotype [48] – among others – in addition to the diet regime [49,50]. Furthermore, physical activity in general and different types of exercise programs can lead to cognitive improvements regardless of baseline cognitive level [51]. The lack of control variables/covariates in these studies makes the relationship between the KD and cognitive functions unclear.

The positive impact of ketones on cognitive function seems to be more noticeable in individuals with impaired glucose metabolism and some degree of cognitive decline [10,19,23,25]. However, studies in healthy participants have shown conflicting results, mainly due to methodological differences and the populations studied. High levels of ketone bodies induced through MCTs appear to enhance cognitive function [23–25]. On the other hand, research on diet-induced ketosis has shown only minor or no changes in cognitive performance [42,43].

On these premises, we explored the effect of the KD on a specific set of cognitive functions in a sample of healthy undergraduate participants, by administering an isocaloric diet whose features were carbohydrate restriction, typically below 30 g per day, rather than exclusively a high-fat content. Expressing undergraduate students’ cognitive potential is fundamental as it constitutes the basis for adequate learning in the university context. Each volunteer who showed interest in the research underwent a screening phase through a medical check-up and an online survey. The medical check-up excluded risk conditions for the participants undergoing the diet. The online survey instead aimed to investigate the psychological chronotype, sleep quality, eating habits, sporting activities, and academic performance. For our exploratory study, we hypothesized that using several cognitive tasks, even more demanding than those used in previous studies and investigating different subcomponents of cognitive functioning, would allow us to more clearly observe diet-induced ketosis’s effects on healthy young adults’ cognitive performance. We aimed to test whether, in addition to marginal improvements in performance on more straightforward cognitive tasks, there was also a change in performance on more demanding cognitive tasks. Indeed, some neuropsychological tests may not be sensitive enough to cognitive changes in healthy participants. We focused on complex executive control functions, including a complex working memory task, the Automated Operation Span Task [52]. Complex span tasks are considered accurate in measuring working memory capacity, and better performance on working memory tasks is related to better academic outcomes [53–55]. Indeed, active maintenance and working on a set of information are essential in forming new concepts and, therefore, for learning processes [53,56]. For these reasons, our study explored the effects of three weeks of KD on a group of healthy undergraduate students with no risks in undertaking the diet, selected in such a way as to minimize differences among participants in aspects such as psychological chronotype, quality sleep, eating habits, academic performance during the last year and sporting activity. Investigating cognitive models associated with particular lifestyle habits and dietary patterns could offer valuable insights into the impact of eating habits on individual characteristics and learning abilities, especially for more complex cognitive tasks.

## Methods

### Participants

Thirty undergraduate student volunteers, who were free from chronic diseases and psychiatric disorders for at least six months before the study, initially participated in the online screening survey. Six participants were excluded due to missing or incomplete information, five due to sleep quality problems, three due to high total anxiety levels, and three due to a decided serotonin or morning chronotype. Additionally, three participants did not show up for the initial cognitive assessment. A total of ten participants took part in the cognitive assessment at T0 before starting the ketogenic diet. Two participants did not attend the second cognitive assessment after three weeks on the ketogenic diet. Thus, the final sample consisted of eight participants. None of the participants reported any side effects related to the dietary treatment.

### Experimental procedure

**Fig 1:**
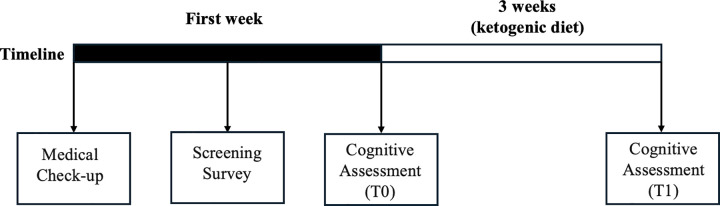
Study timeline.

The study followed a repeated measures design involving cognitive assessments at two time points (T0 or baseline, T1 or follow-up) at the beginning and end of three weeks of an isocaloric ketogenic diet (see Fig 1 and S1 and S2 Tables in S1 File). Each participant who showed interest in the research underwent a screening phase through a medical check-up and an online survey. The medical check-up excluded risk conditions for the participants undergoing the diet. The online survey aimed to investigate the psychological chronotype, sleep quality, eating habits, sporting activities, and academic performance. The project was first advertised via social media and briefings at the University of Foggia. Subsequently, the researchers organized an online meeting to provide all the information on the project to the students who volunteered. The participants who signed the written informed consent underwent a medical examination to verify the absence of risks in embarking on the diet and completed the online questionnaires and the cognitive assessment (T0). After three weeks of the ketogenic diet, the participants underwent the cognitive assessment again (T1). A team of experts provided customized meal plans based on individual preferences, daily calories by macronutrients, and other educational resources for pairing alternative foods. All research activities were conducted in close collaboration between researchers from medical and psychological background respectively from the Department of Clinical and Experimental Medicine and the Department of Humanistic Studies, Letters, Cultural Heritage, and Educational Sciences (University of Foggia). This study procedures were approved by the Ethics Committee of the University of Foggia [Foggia, 10/10/2022, Prot.007/CEpsi] and met the ethical standards of the 1964 Declaration of Helsinki (World Health Organization, 2013). The recruitment period for this study started on April 17, 2023, and ended on July 13, 2023.

### Dietary intervention

The study participants followed an isocaloric ketogenic diet, in which 70–75% of their daily caloric intake derived from lipids and carbohydrate intake was limited to 5–10% (see S1 Table and S2 Table in S1 File). The amount of proteins in the diet was carefully calculated to maintain muscle mass and prevent excess protein from converting into glucose through gluconeogenesis. The mean BMI before and after the intervention was 27.7 and 27.8, respectively (p = 0.68, indicating no statistically significant difference).

Adherence to the diet was checked weekly in the morning under fasting conditions, as ketone levels are known to vary throughout the day depending on dietary intake and physical activity, using Bayer Ketostix (Bayer Leverkunsen, Germany). Dietary ketosis was defined following manufacturer indication, with urine Ketone value higher than 4 mg/L (see S1 Fig in S1 File for individual test).

### Screening survey

We conducted a screening survey using an online software (SurveyMonkey Inc.) to gather information on participants’ eating habits (such as consumption of alcoholic and sugary drinks, intake of caffeine and supplements), sports activities, and academic achievement over the past year. Additionally, participants completed the Morningness-Eveningness Questionnaire (MEQ-SA) [57] to assess their psychological chronotype, the Global Sleep Assessment Questionnaire (GSAQ) [58] to evaluate their sleep quality, and the State-Trait Inventory of Cognitive and Somatic Anxiety (STICSA) [59–62] to measure their anxiety levels. To ensure accurate cognitive performance results, we excluded participants with poor sleep quality (GSAQ total score greater than 30; [58]), those with distinctly morning or serotonin chronotypes (MEQ scores less than 31 and greater than 69; [57]), and participants with high anxiety scores (STICSA total score greater than 42; [63]).

### Assessment of cognitive functions

A neuropsychological battery was administered to the participants to assess cognitive functions (see Table 1) at the beginning (T0) and the end (T1) of the ketogenic diet. The second session for each participant, took place at the same time of day, three weeks after the first session. Participants were tested individually by the same experimenters on weekdays, between 9.00 a.m. and 5.00 p.m.. The procedure was consistent for each participant and lasted about one hour per session. All tasks were completed in paper-and-pencil format, with the exception of the Automated Operation Span Task, administered via the E-Prime software (version 3.0; 2016). To minimize the impact of practice [64,65], alternative forms of the same cognitive tests at T0 and T1 were administered. Additionally, prior to the cognitive assessment (at T0 and T1), participants filled out the State-Trait Inventory of Cognitive and Somatic Anxiety (STICSA).

**Table 1:** Cognitive assessment.

Task	Reference	Cognitive domain functions
Digit Span – **DS**	[66]	Short-term verbal memory
Rey Auditory Learning Test – **RALT**	[67]	Long-term verbal memory
Digit Symbol Test – **DST**	[68]	Attention
Attentional Matrices – **AM**	[69]	Attention
Trail Making Test – **TMT**	[68]	Set-shifting
Stroop Test – **StroopT**	[70]	Cognitive inhibition
Automated Operation Span Task – **Aospan**	[52]	Working memory

### Statistical analysis

We utilized a Bayesian Wilcoxon signed-rank test to analyze the data. The Bayes factor (BF), specifically BF_10_, evaluates how much the observed data supports the alternative hypothesis (H1) over the null hypothesis (H0). Larger values of BF_10_ indicate stronger support for H1. Bayes factors range from 0 to ∞, and a BF_10_ value > 3 suggests moderate support in favor of the alternative hypothesis, while a BF_10_ value > 10 indicates strong support in favor of the alternative hypothesis [71,72]. We used the Bayesian Wilcoxon signed-rank test because it provides a robust non-parametric alternative for paired comparisons in the presence of small sample sizes [73]. In such cases, parametric tests may result in inflated type I or type II error rates [74,75]. Analyses were conducted on raw data with JASP software (version 0.19; JASP Team, 2024).

## Results

Thirty undergraduate students (23 women and 7 men; mean age = 23.7, standard deviation = ± 6.3) completed an online screening survey. Out of these, 8 participants (6 women and 2 men; mean age = 22.9; standard deviation = ± 6.9) completed all phases of the research. This included two cognitive assessments (T0 and T1) conducted immediately before and after three weeks of an isocaloric ketogenic diet. Participants had similar sleep patterns (MEQ score between 31 and 69), low anxiety levels (STICSA score < 42), and good sleep quality (GSAQ score < 30) (see Table 2).

**Table 2:** Descriptive statistics (N = 8).

	Age	MEQ	GSAQ	STICSA(t)
**Mean**	22.9	55.25	22.13	33
**SD**	6.9	8.6	3.27	5.6

*Note*. MEQ = Morningness-Eveningness Questionnaire; GSAQ = Global Sleep Assessment Questionnaire; STICSA = State-Trait Inventory of Cognitive and Somatic Anxiety.

None of the participants engaged in intensive sports activity (< 3 hours a week) or took any medications or supplements. Additionally, participants had similar dietary habits (consuming < 3 sugary and alcoholic drinks per week and < 3 coffees per day) and had passed at least four exams in the last academic year.

As shown in Table 3, participants at T1 performed faster in the DST (number of items reported correctly; BF_10_ = 25.303), the TMT (part A, BF_10_ = 3.931), the StroopT (part B, BF_10_ = 7.115; part C, BF_10_ = 3.184), and in solving the mathematical expressions of the Aospan task (BF_10_ = 45.075), compared to T0. However, accuracy performance was lower in span absolute score (the sum of all correctly recalled set sizes, BF_10_ = 4.080) and in solving mathematical expressions of the Aospan task (BF_10_ = 6.512) at T1 compared to T0 (see Table 3). Finally, state anxiety measured with the STICSA did not differ between T0 and T1 (see Table 4). To confirm the results, the same analyses were recomputed by changing the Bayes Factor (BF_01_; H0 over H1) and formulating alternative hypotheses (i.e., T0 > T1 and T0 < T1). In summary, these analyses confirmed the earlier reported results.

**Table 3:** Bayesian Wilcoxon signed-rank results.

	T0		T1		95% CI		BF_10_
	M	SD	M	SD	Lower	Upper	
DS	6.75	1.58	6.125	1.12	−0.370	1.203	0.621
RALT(i)	47.75	9.16	48.25	10.57	−0.582	0.658	0.347
RALT(d)	11.12	1.88	10.12	2.69	−0.161	1.375	1.160
DST	73.75	11.71	79	12.11	−5.643	−0.371	25.303
AM1(i)	9.75	0.46	9.75	0.46	−0.912	0.945	0.490
AM2(i)	18.87	1.12	18.62	1.59	−0.582	0.699	0.346
AM3(i)	26.62	3.33	27.62	1.40	−0.886	0.386	0.454
AM1(t)	23.68	10.40	22.29	6.86	−0.551	0.670	0.342
AM2(t)	32.31	10.82	29.96	7.97	−0.340	1.004	0.566
AM3(t)	40.36	6.24	37.55	8.31	−0.247	1.116	0.733
TMT(A)	31.17	7.99	25.98	4.22	0.067	1.896	3.931
TMT(B)	52.20	15.39	49.57	16.59	−0.417	0.845	0.457
StroopT(A)	12.75	2.21	11.61	1.55	−0.079	1.388	1.522
StroopT(B)	14.96	1.75	13.75	1.2	0.155	2.052	7.115
StroopT(C)	24.52	6.09	21.44	4.37	0.028	1.627	3.184
Aospan(A)	27.75	19.45	23.5	17.25	0.067	1.862	4.080
Aospan(B)	5.62	3.11	8.37	3.66	−1.973	−0.129	6.512
Aospan(C)	2680.32	1065.97	2121.78	843.05	0.464	3.653	45.075

*Note.* DS = Digit Span; RALT = Ray Auditory Learning Test, (i) immediate, (d) deferred; DST = Digit Symbol Test; AM = Attentional Matrices, (i) item, (t) time; TMT = Trial Making Test, (A) part A, (B) part B; StroopT = Stroop Test, (A) words (B) colored squares, (C) color words in incongruent colors; Aospan = Automated Operation Span Task, (A) Span Absolute Score, (B) Math Errors, (C) Math time.

**Table 4:** Anxiety state scores (STICSA).

	T0		T1		95% CI		BF_10_
	M	SD	M	SD	Lower	Upper	
STICSA	32.37	6.18	30.5	5.15	−0.283	1.082	0.646

## Discussion and conclusion

This study aimed to explore the cognitive effects of an isocaloric ketogenic diet on a group of healthy young adults. The results showed that participants performed the DST, the TMT, and the StroopT and solved mathematical operations in the Aospan task faster at T1 than at T0. However, they performed the Aospan task less accurately. Indeed, participants had a lower absolute span score and solved fewer mathematical expressions correctly at T1 than at T0. Furthermore, participants’ anxiety levels did not differ between baseline and follow-up.

The results of the present explorative study suggest that ketosis may affect the components of cognitive functions differently. First, we found a positive effect of ketosis on visual-motor cognition and processing speed components, as shown by the shorter time required to perform some tests at T1 compared to T0. These results are partly aligned with previous studies [41,42] that reported faster performance in cognitive attention and memory tasks following a low-carbohydrate diet. Several explanations have been proposed for improving cognitive abilities following a ketogenic diet. For example, it has been highlighted that ketone bodies improve the expression of the Brain-Derived Neurotrophy Factor (BDNF) [76]. The BDNF is an essential modulator of the synaptic long-term potentiation (LTP) process related to increased cognitive abilities in adults [77].

In contrast, the diet worsened participants’ accuracy on the Aospan task, suggesting a deterioration of higher cognitive functions in our sample. These results are partly not aligned with Ashton et al. [24], who reported ketosis-induced improvements in cognitive function. However, it is crucial to consider the differences between studies with acute intake of MCTs and studies with dietary protocols. Indeed, the latter could be challenging for participants to follow [78], and an increase in physiological stress following a ketogenic diet has been documented [43]. In this regard, acute corticosteroid administration selectively impairs performance on working memory tasks but not in tasks that assess declarative memory or vigilance tasks with a low working memory load [79].

Moreover, the ketogenic diet induces a metabolic switch from carbohydrate oxidation to fatty acid oxidation. Some human studies suggest that diets high in certain fats may be associated with reduced cognitive performance [80,81]. Other hypotheses have been proposed to explain the negative cognitive changes that diet can induce, including the disruption of neurogenesis [82] and altered membrane and vasculature functioning [83]. However, the mechanisms must still be fully understood [42]. It is also possible that our findings are partly due to the typical side effects that could occur during the ketogenic diet, including nausea and headaches, which could have affected cognitive performance. However, participants did not report any symptoms during the diet. Furthermore, participants were tested after three weeks of the ketogenic diet. Typically, the most prominent negative symptoms occur during the first two weeks of ketogenic adaptation [84,85] or after a prolonged period of ketosis that occurs beyond three weeks [1,86]. Finally, our results are in contrast with those of Shaw et al. [43], who reported no changes in cognitive functions after the ketogenic diet in a small sample of military personnel. In this case, the results of the studies likely differ due to the use of different cognitive tasks. The Aospan task used in the present study requires reporting the correct sequence of letter presentation and solving mathematical expressions simultaneously [52]. The high commitment of cognitive resources required by the task might make it a more sensitive tool for studying cognitive functions in experimental settings with healthy participants. Instead, in the study by Shaw et al. [43], the working memory task used was a standard one-back, which requires remembering the last number presented and reporting whether it is the same as the current one.

It is important to note that the main limitation of the present study is the small study sample. This limitation should be considered when interpreting the results of this study. However, by limiting our participants to healthy, normal-weight individuals free from chronic conditions and obesity and a non-aged sample, we accounted for several important confounding factors in existing studies. Furthermore, we controlled for important psychological and nutritional aspects to exclude further confounding effects on cognitive functions. Finally, it is worth noting that our sample size is similar to other studies investigating cognitive changes in the ketogenic diet [42–44]. A second limitation of the present study is the lack of an isocaloric control group with a different macronutrient distribution. This constitutes a potential confounder, making it difficult to attribute the observed effects exclusively to ketosis. Future studies should therefore employ randomized, isocaloric, and preferably crossover designs to disentangle the specific contributions of macronutrient composition and total energy intake [42,43,78]. An additional limitation could be the lack of information on the participants’ menstrual cycle (being our cohort mostly composed of women (75%)). However, this aspect could be not relevant to our work. Indeed, although experimental evidence on the topic reports neuroimaging-detectable changes in brain activation and connectivity during the menstrual cycle [87], the effects of menstrual cycle phases on cognitive function reported in the literature are mixed [87,88], and a recent meta-analysis [89] did not observe any systematic and robust evidence of significant changes in cognitive performance due to menstrual cycle phases. Future studies should measure and control for the menstrual cycle phase (ideally with hormonal confirmation) or stratify analysis accordingly. Furthermore, although participants who completed the dietary treatment did not report any side effects, we observed a high dropout rate among participants. This pattern is consistent with previous reports of adherence challenges in ketogenic diet interventions [90]. It is therefore possible that participants who did not attend the second cognitive assessment experienced difficulties in adhering to the dietary plan.

Based on our findings, future studies should investigate different components of cognitive functions. In addition to the dissociation between visual-motor and cognitive components, both explicit and implicit aspects of cognitive functions should be investigated. Evidence of a dissociation between performance on implicit/explicit tasks about chronotype is reported in the literature [91–93], as well as an important relationship between ketogenic diet, chronobiology, and cognitive functions [94–96]. Furthermore, the role of stress in participants in dietary studies should be further investigated through self-reported and physiological measures concerning the diet. Stress can affect cognitive functions [97], and increases in physiological stress have been reported during a ketogenic diet [43]. Moreover, participants’ motivations for dieting should be investigated. Approaching a diet as an athlete, doing it for weight loss, or doing it within a university study context could influence the observed results.

Our results highlighted that the ketogenic diet impacted cognitive functions by improving the visuomotor and processing speed components. However, it also worsened the accuracy of a complex working memory task. Regarding this last point, it is known that individuals who perform well in working memory tasks can stay better focused on the task and that a good working memory is essential for learning processes [53,98]. For these reasons, our results could have important implications for research and learning contexts.

## Supporting information

S1 File Individual test, Ketogenic diet food plan for women and men. (PDF)
