# Supplementary material for: Faster but less accurate: An explorative study on the effects of three weeks of ketogenic diet on cognitive functions in undergraduate students
Source: PLoS One. 2026 Jan 14;21(1):e0338877. doi: 10.1371/journal.pone.0338877 (PMC12803453; doi:10.1371/journal.pone.0338877)
Supplement: S1 File — (PDF) [file pone.0338877.s001.pdf]

Fig. S1

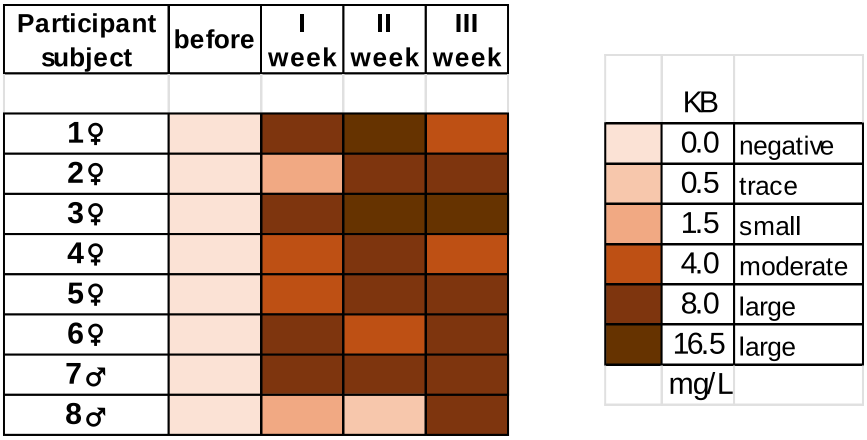

Figure legend: The assay was performed by test strips as described in the *Methods* section of the main text. The results refer to the test performed before the administration of the ketogenic diet and at the ends of the indicated weeks following the ketogenic regimen. The colour codes refer to those indicated by the strip manufacturer. ♀, female; ♂, male.

Table S1-KETOGENIC DIET FOOD PLAN FOR WOMEN (2200 KCAL)

MEAL	FOOD	QUANTITY	ALTERNATIVES
**BREAKFAST**	Whole milk yogurt	250 g	Whole milk 250 g, Greek yogurt 250 g
	Walnuts	20 g	20 g almonds, pistachios, cashews, peanuts (without added salt)
	Rusks	15 g	10 g oat flakes or puffed rice<br>10 g whole grain corn flakes<br>10 g muesli
	Butter	20 g	25 g cream or mascarpone<br>10 g avocado<br>20 g margarine or vegetable butter
**SNACK**	Protein bar	40 g	-
	Dark chocolate (>70%)	40 g	40 g peanut butter<br>30 g nuts
**LUNCH**	Chicken breast	150 g	2 eggs<br>150 g lean meat: beef, veal, white meat like poultry, rabbit<br>200 g fish and seafood<br>170 g canned tuna, mackerel or salmon in water<br>130 g cooked ham, prosciutto, speck, bresaola<br>70 g cooked or cured ham + 1 egg<br>200 g tofu or seitan
	Extra virgin olive oil	40 g	-
	Vegetable side dish *****	100 g	-
**SNACK**	Almonds	40 g	40 g walnuts, pistachios, cashews, peanuts (without added salt)
**DINNER**	Cod	150 g	2 eggs<br>100 g lean meat: beef, veal, white meat like poultry, rabbit<br>150 g fish and seafood<br>120 g canned tuna, mackerel or salmon in water<br>100 g cooked ham, prosciutto, speck, bresaola<br>50 g cooked or cured ham + 1 egg<br>150 g tofu or seitan
	Extra virgin olive oil	40 g	-
	Vegetable side dish	100 g	-
	Mayonnaise	30 g	30 g other types of sauces

Table S2 -KETOGENIC DIET FOOD PLAN FOR MEN (2400 KCAL)

MEAL	FOOD	QUANTITY	ALTERNATIVES
**BREAKFAST**	Whole milk yogurt	300 g	Whole milk 300 g, Greek yogurt 300 g<br>Soy drink or plant-based beverages 300 g
	Walnuts	25 g	25 g almonds, pistachios, cashews, peanuts (without added salt)
	Rusks	15 g	10 g oat flakes or puffed rice<br>10 g whole grain corn flakes<br>10 g muesli
	Butter	20 g	25 g cream or mascarpone<br>10 g avocado<br>20 g margarine or vegetable butter<br>15 g lard
**SNACK**	Protein bar	40 g	-
	Dark chocolate (>70%)	40 g	40 g peanut butter<br>30 g nuts
**LUNCH**	Chicken breast	250 g	3 eggs<br>250 g lean meat: beef, veal, white meat like poultry, rabbit<br>300 g fish and seafood<br>280 g canned tuna, mackerel or salmon in water<br>220 g cooked ham, prosciutto, speck, bresaola<br>110 g cooked or cured ham + 1 egg<br>300 g tofu or seitan
	Extra virgin olive oil	40 g	-
	Vegetable side dish*	100 g	-
**SNACK**	Almonds	50 g	50 g walnuts, pistachios, cashews, peanuts (without added salt)
**DINNER**	Cod	200 g	2 eggs<br>150 g lean meat: beef, veal, white meat like poultry, rabbit<br>200 g fish and seafood<br>170 g canned tuna, mackerel or salmon in water<br>170 g cooked ham, prosciutto, speck, bresaola<br>70 g cooked or cured ham + 1 egg<br>200 g tofu or seitan
	Extra virgin olive oil	40 g	-
	Vegetable side dish	100 g	-
	Mayonnaise	30 g	30 g other types of sauces

*Asparagus, Eggplant, Swiss chard, Broccoli, Celery, Mushrooms, Cauliflower, Radishes, Watercress, Cucumbers, Green salad, Endive or Belgian lettuce, Spinach, Fennel, Radicchio, Soy sprouts, Cardoon, Green peppers, Arugula, Savoy cabbage, Zucchini, Artichokes, Cabbage, Brussels sprouts, Green beans, Turnips, Leeks, Red and yellow peppers, Tomatoes
